# Distal radius fractures reduction and immobilization using a novel device in older adults in emergency department

**DOI:** 10.3389/fsurg.2026.1747947

**Published:** 2026-09-03

**Authors:** Dongdong Jiang, Junwei Yan, Xiao Wang, Bin Liang, Zhaowei Yin

**Affiliations:** 1 Department of Orthopedics, Nanjing First Hospital, Nanjing Medical University, Nanjing, China; 2 Department of Orthopedics, Nanjing Qixia District Hospital, Nanjing, China

**Keywords:** bone, displacement, distal radius, fracture, treatment outcome

## Abstract

**Background:**

The purpose of this study was to introduce a new device for the reduction and immobilization of distal radius fractures.

**Methods:**

We retrospectively reviewed 40 patients aged 65 years or older who had been treated for displaced distal radius fractures between January 1 and December 31, 2023. Patients were grouped according to the treatment documented in their medical records: conventional manual closed reduction and cast immobilization (*n* = 20) or device-assisted reduction and cast immobilization (*n* = 20). Clinical, radiographic, and patient-reported outcomes recorded at 1 week, 6 months, and 1 year were extracted and compared.

**Results:**

The groups had equal distribution in age, gender, injured side, classification of fracture. Volar tilt (Vt), and Ulnar variance (Uv) were better in device group at each follow-up point, Radial inclination (Ri) and Radial length (Rl) were similar at 6-month and 1-year follow-up. Wrist flexion, extension and pronation were better in device group at 6-month and 1-year follow-up. DASH scores were better in device group at 6-month follow-up and similar at 1-year follow-up. Skin damage, neurovascular injury and tenosynovitis did not occur in any patients.

**Conclusion:**

The device is convenient and effective for reduction of distal radius fractures. For our future work, we propose to improve the device to incorporate control by artificial intelligence and to reduce the size and weight to more accurately and intelligently control the device and make it easier to carry and operate.

## 1 Introduction

Distal radius fractures (DRFs) are commonly observed in emergency and orthopedic services, accounting for up to 15% of all extremity fractures (1). Young patients usually suffer from high-energy injuries, while DRFs in elderly patients often result from low-energy trauma (2). DRFs include closed reduction and cast stabiliza tion, percutaneous Kirschner wire fixation, external fixation, and open reduction and internal fixation (ORIF) (3). Nevertheless, closed reduction and cast immobilization remains a major treatment approach for DRFs, particularly in older adults (4, 5). These approaches rely heavily on operator skill and are associated with a non-trivial risk of loss of reduction and need for re-intervention. Common limitations are variable traction control, difficulty in restoring and maintaining palmar tilt, radiation exposure during fluoroscopic checks, and the requirement for multiple operators in some maneuvers (6). We developed and evaluated a novel reduction device capable of delivering controlled, continuous traction and multiplanar correction during closed reduction procedures.

In this retrospective comparative cohort study, we compared radiographic and functional outcomes documented after device-assisted reduction and conventional manual reduction in adults aged 65 years or older. The outcomes of interest were early radiographic alignment (volar tilt and ulnar variance) and redisplacement, wrist range of motion, DASH score, and complications.

## 2 Materials and methods

### 2.1 Retrospective study design and patient selection

We retrospectively reviewed the medical records and imaging data of patients with DRFs treated between January 1 and December 31, 2023. Patients were identified from institutional clinical records and classified according to the reduction method they had received during routine care. Twenty patients had undergone conventional manual closed reduction and cast immobilization, and 20 had undergone device-assisted reduction and cast immobilization. The inclusion criteria were as follows: patients with closed, displaced DRFs (AO/ATO A1∼A3, C1∼C3); patients aged ≥65 years; patients with injury time within 24 h. The exclusion criteria were as follows: patients with open fractures, patients aged < 65 years, patients with fractures older than 24 h, patients with fractures associated with radiocarpal joint dislocation or subluxation. All patients provided written informed consent.

### 2.2 Design of the device

**Figure 1:**
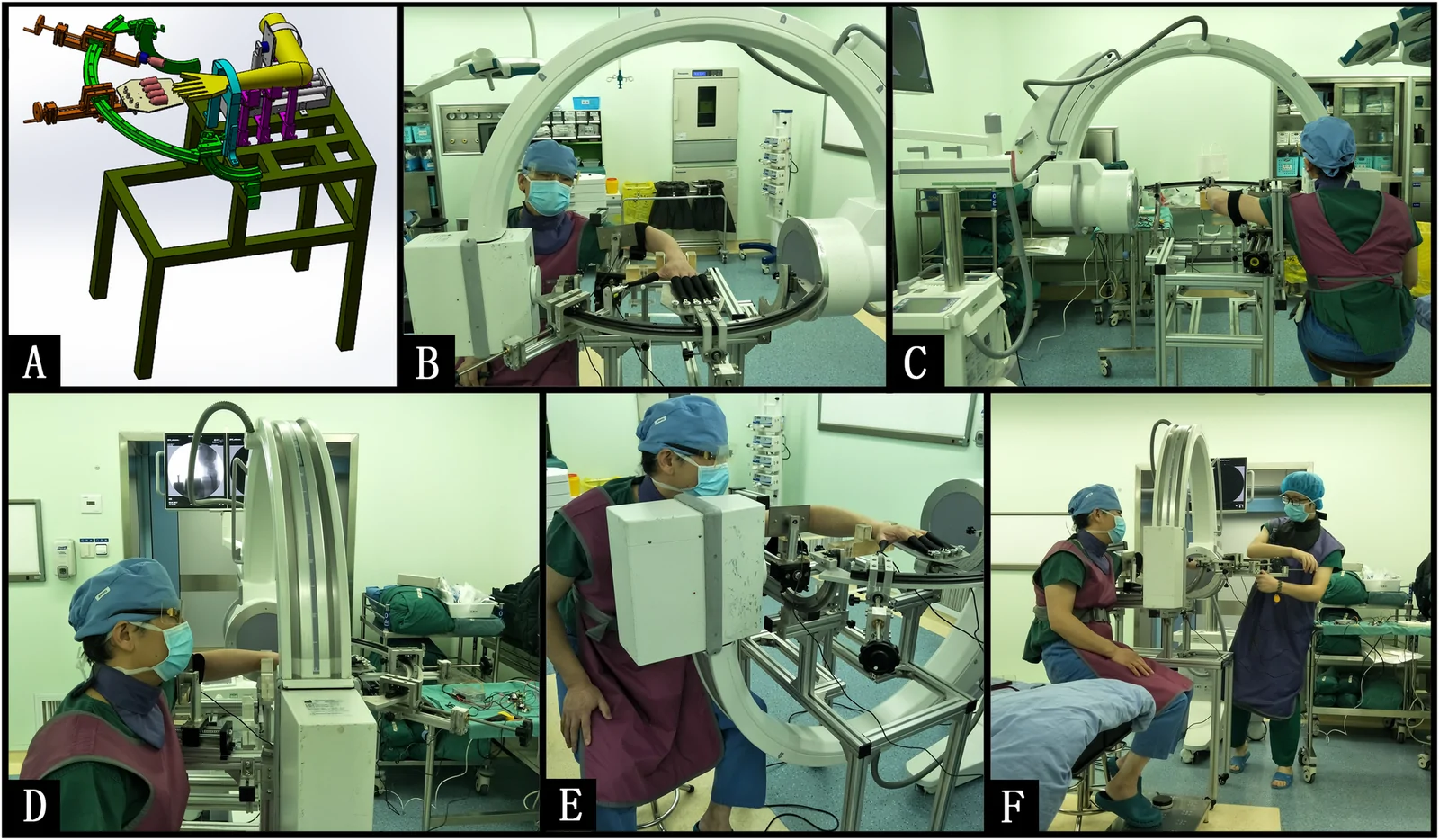
**(A)** the overall framework of the fracture reduction device for distal radius. **(B–E)** The position of patient and C-arm fluoroscopy was experimented by clinicians. **(F)** The reduction procedures were performed by another doctor by operating the device.

The device we have developed was with the help of the team lead by professor Wang from the School of Mechanical Engineering, Southeast University. The main components are: (1) an adjustable upper-arm fixation frame that secures the humerus and minimizes shoulder motion; (2) an elbow fixation unit with a pivot to lock elbow flexion angle; (3) a double-plane curved guide rail with two movable sliders (horizontal and vertical) that allow controlled ulnar/radial deviation and flexion/extension respectively; (4) a finger-trap style traction assembly that applies longitudinal traction to the thumb and the 2nd–5th digits; (5) a wrist rotational calibration device to align the device axes with the patient's anatomical axes; and (6) hand grips and mechanical locking screws for fine adjustment. Unlike conventional finger-trap systems, the principal innovation is the combination of sustained traction with independent multiplanar correction and position locking during cast molding. The device permits independent adjustment of traction magnitude, ulnar/radial deviation, and flexion/extension. All metallic parts are medical-grade stainless steel or anodized aluminum; soft contact interfaces use padded medical silicon to avoid pressure points. Detailed engineering drawings and prototype specifications have been reported previously (7) and are summarized in Figure 1.

### 2.3 Reduction technique

**Figure 2:**
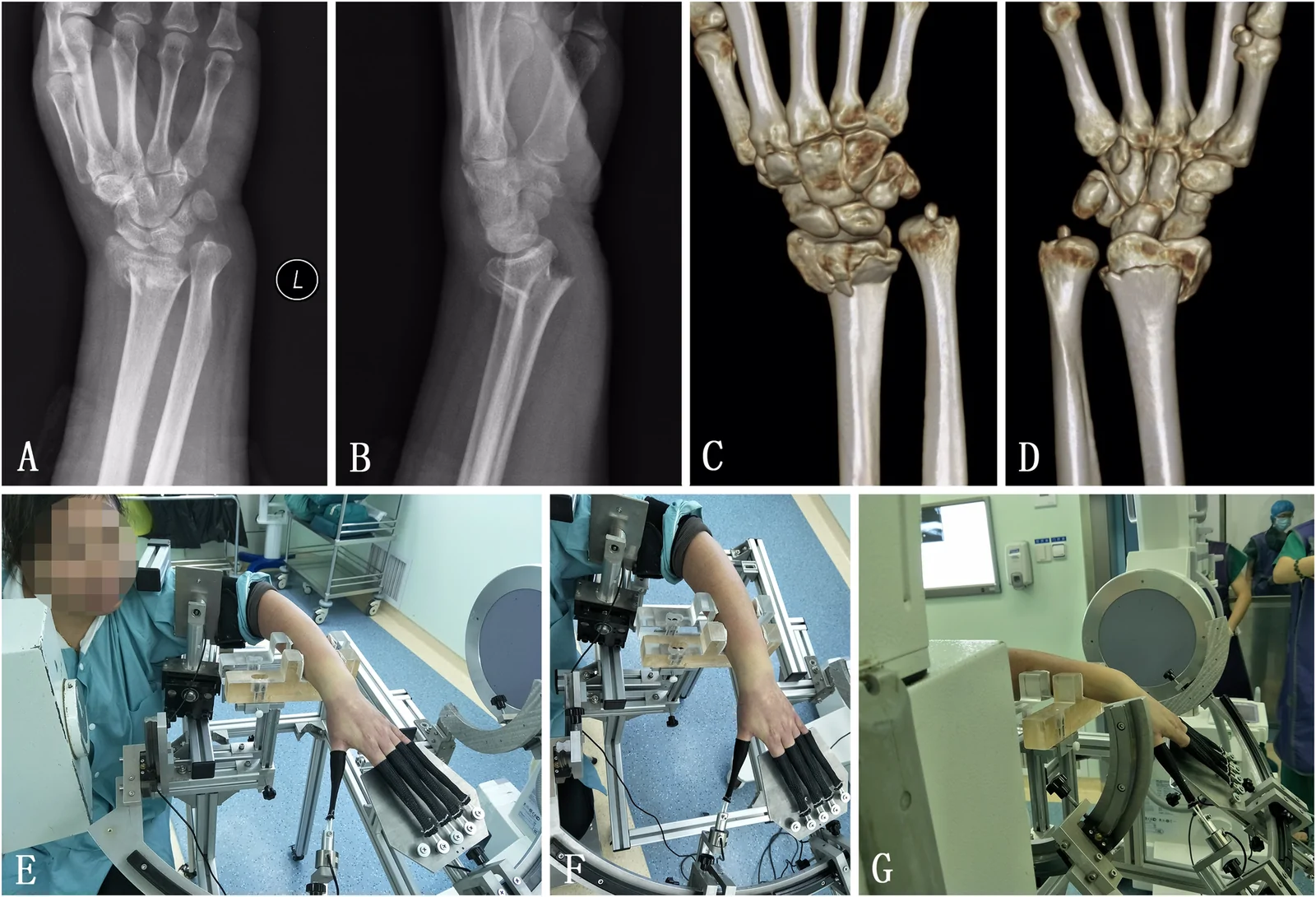
A 66-year old female fell down from a standing height and suffered from fractures of left distal radius and ulnar styloid fractures. **(A–D)** The x-ray and CT views of the fractures. **(E–G)** The position of patient and the injured upper limb.

**Figure 3:**
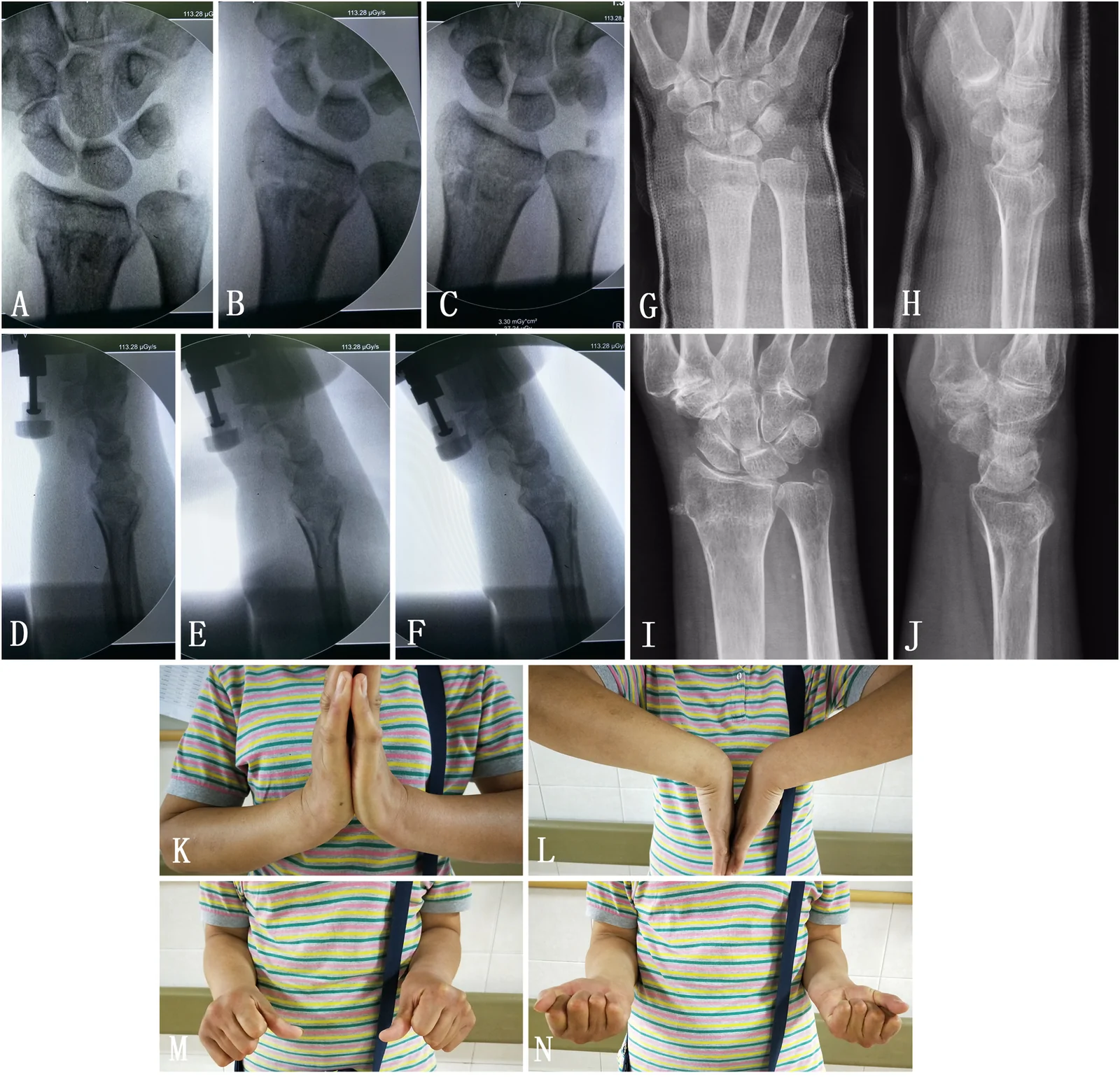
**(A–C)** the anteroposterior fluoroscopy image of fracture site when the distal fragment was gradually reduced by traction and ulnar deviation by operating the device. **(D–F)** The fluoroscopy image of fracture site when the distal fragment was gradually reduced by flexion by operating the device. **(G–H)** The immediate x-ray of fracture after reduction and cast immobilization. **(I,J)** The x-ray of fracture at 6-month follow up. **(K–N)** The ROM of injured wrist at 6-month follow-up.

**Figure 4:**
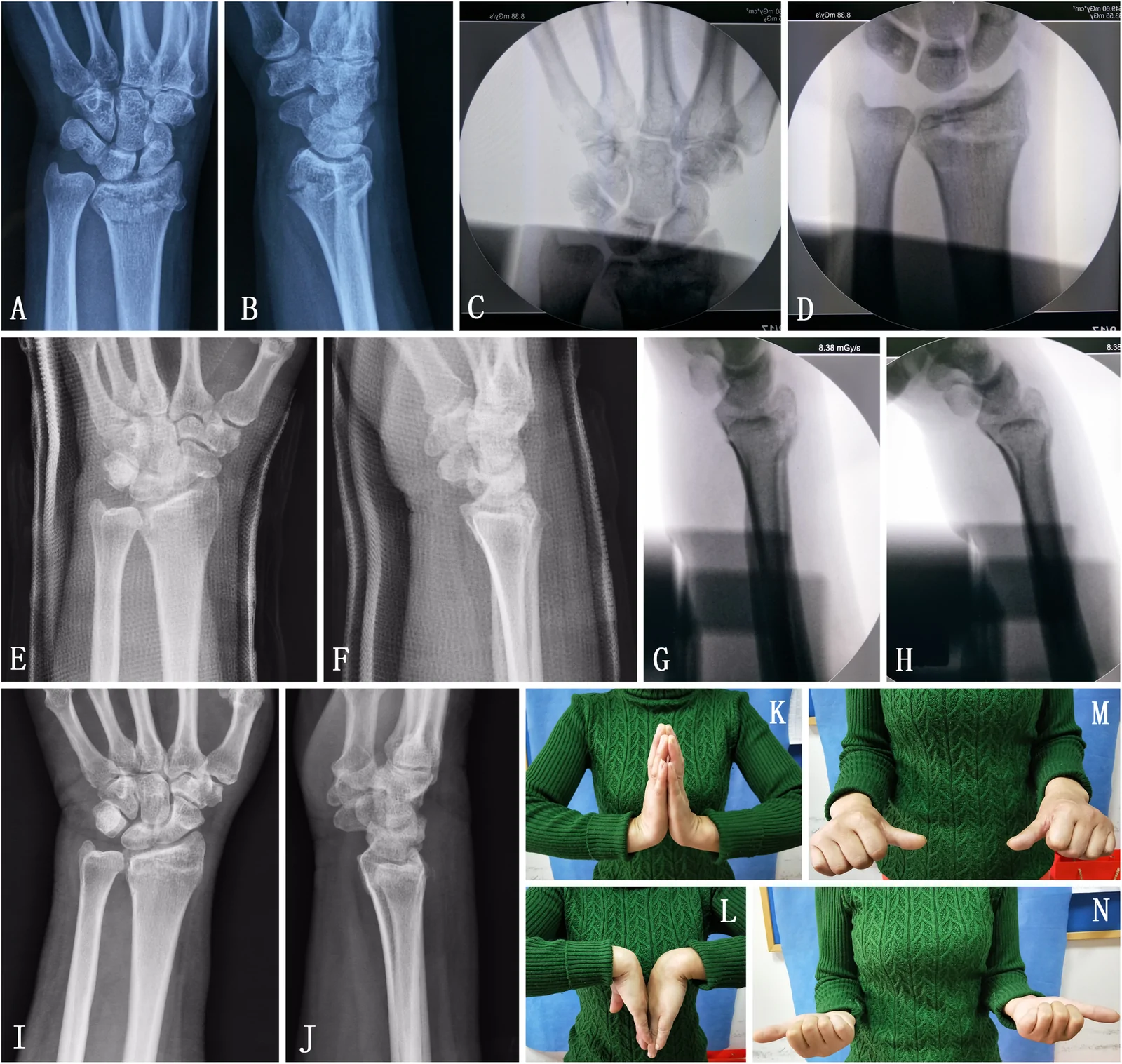
A 70-year old female fell down from a standing height and suffered from fracture of left distal radius. **(A,B)** x-ray views of the fracture. **(C,D)** The anteroposterior fluoroscopy image of fracture site when the distal fragment was gradually reduced by traction and ulnar deviation by operating the device. **(G,H)** The fluoroscopy image of fracture site when the distal fragment was gradually reduced by flexion by operating the device. **(E,F)** The immediate x-ray of fracture after reduction and cast immobilization. **(I,J)** The x-ray of fracture at 6-month follow up. **(K–N)** The ROM of injured wrist at 6-month follow-up.

All patients were sitting on a chair and received a hematoma block. In conventional group, traction was applied with manual traction and countertraction, hereafter, a reduction maneuver was performed as described by Charnley (8). Briefly, two doctors applied manual traction and countertraction by hands, and increasing the deformity by dorsiflexing, then applying volar direction press and palmar flexion of the wrist. Next, pronated the wrist and forced into ulnar deviation to locking the reduction. Finally, the plaster of paris was molded for a three point fixation in approximately 30° extension of the wrist. In device group, the upper arm of patient was fixed by the upper arm fixation device, and the elbow was attached to the elbow fixation device. Then, the surgeon raised one of the double forearm supporting devices to provide support for the patient's forearm and used the calibration device at the wrist rotational axis to ensure the patient's wrist rotational axis of flexion/extension and rotational axis of radial/ulnar deviation were consistent with the rotational axis of the double-plane curved guide rail. Next, the surgeon used the finger-trap mechanism of the thumb and 2th∼5th fingers to stretch the patient's thumb and the other four fingers to disimpact the fracture, and the stretch was maintained for several minutes to release the intercalated fracture and the surrounding muscles and ligaments. After the initial procedures were prepared, the doctor controlled the device for the next reduction steps. First, the horizontal sliders were moved on the double-plane curved guide rail to keep the wrist in a slight ulnar deviation state. Second, the surgeon moved the vertical sliders on the double-plane curved guide rail to move the wrist and maintain a state of flexion or extension. All reduction procedures were monitored in a fluoroscopy intensifier. Next, a below-elbow forearm cast was applied to maintain the position of the wrist, with care taken not to immobilize the metacarpophalangeal joint. The supports maintained traction and reduction but could be repositioned before final locking to permit molding. Once the cast had hardened sufficiently to maintain the reduction, traction was released, the finger traps and supports were removed sequentially, and final fluoroscopic images were obtained. If alignment was unsatisfactory, the cast was removed and the procedure was repeated. The device was not incorporated into the cast and did not remain attached after casting. (Figures 2–4).

### 2.4 Postoperative management and follow-up evaluation

Follow-up data were extracted retrospectively from clinical records and archived radiographs. The institutional post-reduction protocol included finger exercises during immobilization, clinical and radiographic evaluation at 1 week, weekly examinations for the next 3 weeks, cast removal at 6 weeks, and subsequent wrist exercises. Acceptable alignment was defined as less than 10 degrees of residual dorsal angulation from neutral, less than 2 mm of ulnar variance relative to the contralateral side, no more than 1 mm of articular step-off, no dorsal or volar distal radioulnar joint subluxation on a true lateral radiograph, and no distal radioulnar joint widening on the anteroposterior radiograph. Radial inclination, radial length, and volar tilt were extracted from radiographic assessments (9). Available ROM and DASH data recorded at 6 months and 1 year were collected. Radiographic and functional outcomes had been assessed by a surgeon who was not involved in the reduction procedures; however, because group assignment could be inferred from the clinical records and imaging workflow, blinded outcome assessment could not be confirmed.

### 2.5 Statistical analysis

Analyses were performed with SPSS version 25.0 for Windows (SPSS Inc., Chicago, IL, USA). Categorical variables were compared using the chi-square test or Fisher exact test, as appropriate. Normally distributed continuous variables are reported as mean ± standard deviation and were compared using independent-samples t tests. Median (interquartile range) were compared using the Mann–Whitney U test. Range-of-motion and radiographic measurements were compared between groups at each follow-up point using independent-samples t tests. Because this was a retrospective study using an available cohort, no *a priori* sample-size calculation was performed and the analyses are exploratory. No propensity-score adjustment or multivariable control for treatment-selection confounding was performed; effect estimates should therefore be interpreted as associations rather than causal effects. A two-sided *p* value <0.05 was considered statistically significant.

## 3 Results

### 3.1 Patient characteristics

Patient characteristics are shown in Table 1. Forty participants were included, with 20 in the conventional group and 20 in the device group. In the conventional group, 16 (80%) were female and 4 (20%) were male; mean age was 68.7 years (range, 65–74). Twelve fractures (60%) involved the left wrist and 8 (40%) the right; 11 were AO type A and 9 were type C. In the device group, 15 participants (75%) were female and 5 (25%) were male; mean age was 69.1 years (range, 65–75). Eleven fractures (55%) involved the left wrist and 9 (45%) the right; 10 were type A and 10 were type C. Age, sex, injured side, and fracture classification were similar between groups (Table 1). Time from injury to reduction did not differ significantly. Median preparation time was longer in the device group than in the conventional group (7 vs. 3 min), although actual reduction and cast-immobilization time did not differ significantly. All patients received a hematoma block before reduction (Table 1).

**Table 1:** General information of the patients of the two groups.

Characteristic	Conventional group	Device group	*P* value
Age(years)	68.70 ± 2.79 (65∼74)	69.10 ± 3.14 (65∼75)	0.673
Gender	16F/4M	15F/5M	0.075
Hand dominance	12L/8R	11L/9R	0.749
OTA/AO classification	11A/9C	10A/10C	1.000
Time from injury to reduction (h)	3.00 (2.00，4.75)	3.00 (2.00，4.00)	0.687
Time of preparing reduction (min)	3.00 (2.25，4.00)	7.00 (6.00，7.00)	<0.05
Time of reduction and immobilization (min)	10.00 (10.00，11.00)	10.50 (10.00，11.00)	0.966

### 3.2 Radiologic results

Immediately post reduction and at 1-week follow-up, the device group achieved significantly improved volar tilt (Vt) and ulnar variance (Uv) compared with the conventional group (post reduction Vt: device 9.15 ± 3.12° vs. conventional 6.4 ± 7.45°, *p* = 0.0168); 1 week Vt: device 7.35 ± 3.93° vs. conventional 3.2 ± 9.08°, *p* = 0.0044; post reduction Uv: device 0.14 ± 0.21° vs. conventional 0.28 ± 0.59°, *p* = 0.0102, 1 week Uv: device 0.36 ± 0.32° vs. conventional 0.43 ± 0.73°, *p* = 0.0043). while radial length (Rl) was similar in two groups immediately after reduction and at 1-week follow-up. At 6 months and 1 year, Vt and Uv remained better in the device group (6 months Vt: device 7.1 ± 3.7° vs. conventional 3.05 ± 8.79°, *p* = 0.0036); 1 year Vt: device 6.85 ± 3.99° vs. conventional 2.95 ± 9.12°, *p* = 0.0074; 6 months Uv: device 0.37 ± 0.33° vs. conventional 0.54 ± 0.82°, *p* = 0.0079, 1 year Uv: device 0.42 ± 0.32° vs. conventional 0.56 ± 0.79°, *p* = 0.0152).; Ri and Rl were similar between groups at later follow-up (Table 2).

**Table 2:** Radiographic parameters of two groups.

Radiographic parameter	Conventional group	Device group	*P* value
Pre-reduction			
volar tilt (°)	−12.25 ± 4.97	−12.45 ± 2.82	0.8166
radial inclination (°)	13.6 ± 4.35	13.95 ± 2.20	0.6232
radial length (mm)	6.65 ± 1.81	6.4 ± 0.92	0.404
ulnar variance (mm)	3.05 ± 1.49	3.1 ± 0.77	0.8392
Post-reduction			
volar tilt (°)	6.4 ± 7.45	9.15 ± 3.12	0.0168
radial inclination (°)	19.95 ± 4.37	21 ± 2.27	0.037
radial length (mm)	9.89 ± 1.85	9.7 ± 0.9	0.6123
ulnar variance (mm)	0.28 ± 0.59	0.14 ± 0.21	0.0102
1 week post-reduction			
volar tilt (°)	3.2 ± 9.08	7.35 ± 3.93	0.0044
radial inclination (°)	18.75 ± 4.00	20.7 ± 2.24	0.0079
radial length (mm)	8.9 ± 2.49	9.15 ± 1.19	0.5273
ulnar variance (mm)	0.43 ± 0.73	0.36 ± 0.32	0.0043
6 months post-reduction			
volar tilt (°)	3.05 ± 8.79	7.1 ± 3.7	0.0036
radial inclination (°)	18.68 ± 3.96	19.95 ± 2.40	0.0506
radial length (mm)	8.89 ± 2.14	9.05 ± 1.28	0.5126
ulnar variance (mm)	0.54 ± 0.82	0.37 ± 0.33	0.0079
1 year post-reduction			
volar tilt (°)	2.95 ± 9.12	6.85 ± 3.99	0.0074
radial inclination (°)	18.21 ± 3.43	19.2 ± 2.62	0.1195
radial length (mm)	8.79 ± 2.30	9.05 ± 1.20	0.5118
ulnar variance (mm)	0.56 ± 0.79	0.42 ± 0.32	0.0152

### 3.3 Functional results

At 6 months and 1 year follow-up, mean wrist flexion was greater in the device group (6 months: 59.75 ± 6.42°, 1 year: 63.5 ± 5.94°) than in the conventional group (6 months: 50.25 ± 6.80°, *p*=<0.001, 1 year: 57.75 ± 6.42, *p* = 0.0067°). Similar between-group advantages were observed for extension (6 months: device 59.5 ± 6.87° vs. conventional 53.25 ± 8.84°, *p* = 0.0198; 1 year: device 68.3 ± 4.73° vs. conventional 62.25 ± 6.98°, *p* = 0.0034) and pronation (6 months: device 58.75 ± 8.2° vs. conventional 49.75 ± 6.22°, *p* = 0.0005; 1 year: device 66.25 ± 4.44° vs. conventional 60.25 ± 5.12°, *p* = 0.0004) (Table 3). Supination did not differ significantly between groups at any time point. The mean DASH score at 6 months was lower in the device group (26.13 ± 8.03) than in the conventional group (32.79 ± 10.38; *p* = 0.0329). Although this between-group difference was statistically significant, its clinical importance should be interpreted cautiously because reported MCID estimates for the DASH vary across studies (10). At 1 year, the mean DASH score remained numerically lower in the device group, but the difference was not statistically significant (14.21 ± 4.74 vs. 17.38 ± 6.22, *p* = 0.0856) (Table 3).

**Table 3:** Functional parameters of two groups.

Functional parameter	Conventional group	Device group	*P* value
6 months			
Extension (°)	53.25 ± 8.84	59.5 ± 6.87	0.0198
Flexion (°)	50.25 ± 6.80	59.75 ± 6.42	<0.001
Supination (°)	78.75 ± 5.89	81.5 ± 5.5	0.1451
Pronation (°)	49.75 ± 6.22	58.75 ± 8.2	0.0005
DASH	32.79 ± 10.38	26.13 ± 8.03	0.0329
1 year			
Extension (°)	62.25 ± 6.98	68.3 ± 4.73	0.0034
Flexion (°)	57.75 ± 6.42	63.5 ± 5.94	0.0067
Supination (°)	83.25 ± 3.63	85.5 ± 3.84	0.0713
Pronation (°)	60.25 ± 5.12	66.25 ± 4.44	0.0004
DASH	17.38 ± 6.22	14.21 ± 4.74	0.0856

### 3.4 Complications

**Figure 5:**
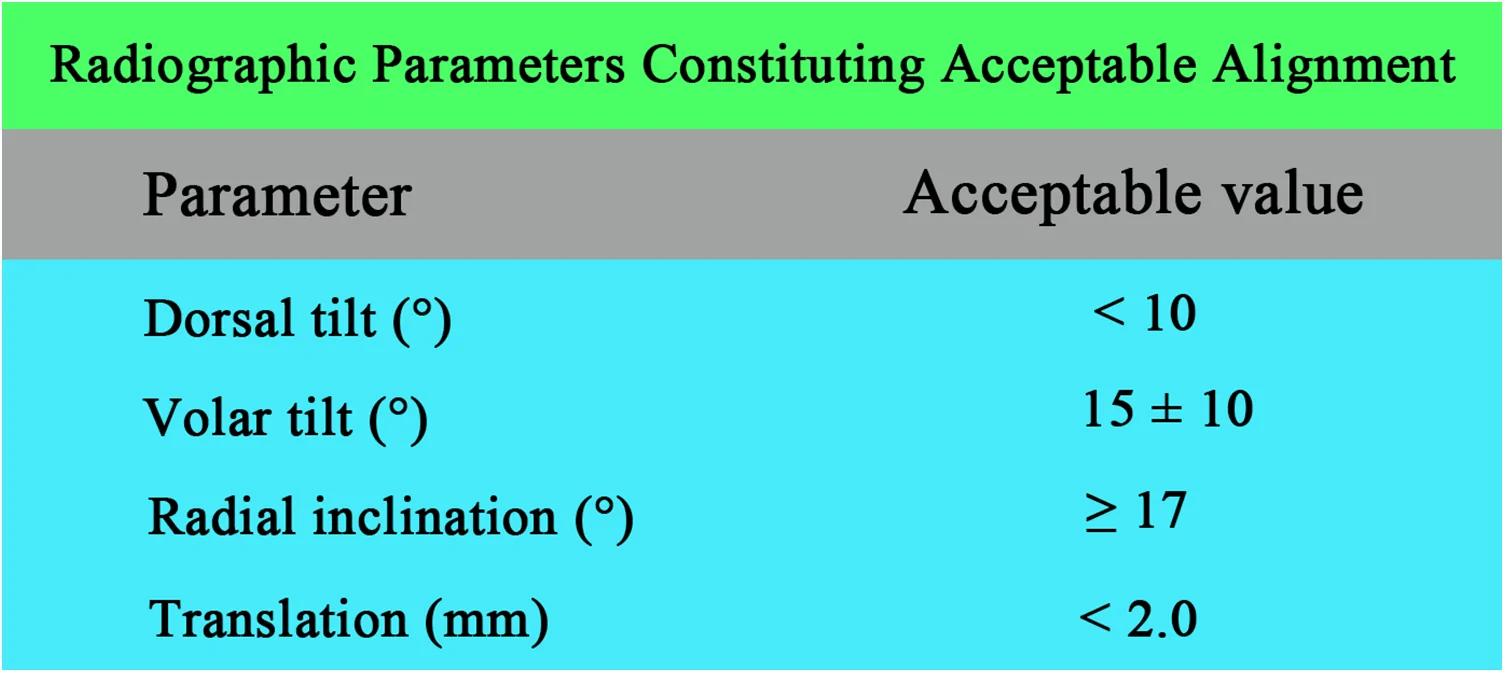
Radiographic parameters constituting acceptable alignment.

In the conventional group, four patients experienced cast-related discomfort, which was managed by cast modification at the first-week follow-up. Eight patients required cast replacement at the second week due to loosening after the resolution of swelling. Secondary displacement occurred in seven of twenty patients (35%) during the first week of follow-up. According to the guidelines of the American Academy of Orthopaedic Surgeons (AAOS) (11), surgical treatment was indicated for three of these patients; however, these patients declined surgery and continued conservative management with cast immobilization until fracture healing. In the remaining four patients, radiographic parameters deteriorated compared with immediate post-reduction values but remained within acceptable limits (Figure 5) (12). These patients also continued cast immobilization until union. The mean duration of cast application was 6.2 weeks (range, 5–8 weeks). In the device group, three patients reported cast discomfort that was alleviated through modification at the first-week follow-up. Six patients required cast replacement at the second week after swelling subsided. Secondary displacement was observed in two of twenty patients (10%) at the first-week follow-up; however, all radiographic parameters remained within acceptable criteria, and cast immobilization was maintained until fracture healing. The mean duration of immobilization was 6.1 weeks (range, 5–8 weeks). No cases of skin damage, neurovascular injury, or tenosynovitis were observed in either group.

## 4 Discussion

DRFs are among the most common fractures (13), and nonoperative management by closed reduction and immobilization remains important for many older patients (14). The relationship between radiographic alignment and long-term function is debated (15, 16), although other studies support an association between anatomic restoration and outcome (17, 18). Akar and Yücel likewise reported a positive association between radial inclination and Mayo wrist scores after fixation of intra-articular DRFs (19). In the present study, device assistance improved early alignment and 6-month function, whereas the between-group DASH difference was no longer statistically significant at 1 year. The early benefit may nevertheless be clinically meaningful in older adults because earlier recovery of wrist motion and upper-limb function may facilitate independence in dressing, hygiene, transfers, and use of walking aids during the period when vulnerability is greatest.

The conventional method of closed reduction was manual manipulation by 2 doctors standing opposite of each other and using the traction-countertraction method to produce and maintain wrist traction. Another assistant should prepare and mold the plaster cast (14). Currently, in China, the closed reduction of DRFs is almost accomplished using conventional techniques, especially in basic-level hospitals. In our opinion, although doctor experience and the specific maneuvers are important for the reduction technique, there still remain several disadvantages. First, the force of traction needed in each patient is different. Without the image intensifier, whether the radial length or height was restored is unknown. When the patient is rechecked through x-ray after the plaster splint is molded, if the reduction is unsatisfactory, the fracture may need re-reduction, which will add pain and anxiety to patients and make the second reduction more difficult, as Schermann et at (20). revealed that a second closed reduction worsened dorsal comminution and had a negative effect on fracture stability. Second, when the assistant prepares the plaster cast, doctors should maintain the position of the fractured limb, which must sometimes be accomplished by one doctor alone. However, after a long time of traction-countertraction, especially in obese or strong patients, it is difficult to maintain the strength at the same level, leading to a loss of reduction of the fracture site.

Mechanical traction devices for DRFs are not new. Shah et al. described a looped-stockinette technique (21), Salvi described a handshake technique (22), and Wise et al. reported a traction jig using finger traps and an 8-kg counterweight (23). A systematic review comparing finger-trap traction with manual traction found different radiographic strengths for the two methods and did not establish a single optimal technique (24). The device specific contribution is the integration of sustained longitudinal traction with a calibrated double-plane guide rail that independently controls ulnar/radial deviation and flexion/extension and locks the corrected position during cast molding. Because treatment was selected during routine care rather than assigned randomly, the observed differences may also reflect baseline clinical factors that were not captured in the records.

The device enabled one physician to perform preparation, reduction, and cast immobilization while maintaining stable multiplanar control. It can be used in an emergency department or operating room, permits fluoroscopic observation and incremental correction without relying on sustained operator strength, and may be suitable for clinicians with less experience after appropriate training. These features could be useful in busy emergency services and settings with limited staffing. However, setup required a median of approximately 4 additional minutes in this study (7 vs. 3 min). Whether reduced personnel requirements, fewer repeat reductions, and lower redisplacement offset this setup time requires formal workflow and cost-effectiveness evaluation. No skin lesion, neurovascular injury, or tenosynovitis was observed in either group.

In clinical practice, manual manipulation is widely used. In the United Kingdom, fractures are usually reduced by manual manipulation with countertraction, while in the United States, Colles’ fractures are often reduced with the use of finger traps (25). However, independently using finger trap for traction and reduction was not enough, finger trap traction cannot reduce the fracture in both planes, and palmar tilt is difficult to restore by finger trap traction alone. longitudinal traction could not restore and preserve anatomical palmar tilt. That is, both accurately controlling the traction-countertraction force and manually manipulating the fragment angle were rather difficult. However, this device provides a novel field for doctors to adjust treatment parameters in order to precisely manage the problems.

The current reduction device permits force control in three planes (axis, horizontal, and sagittal planes), and the position of the hand and wrist can be adjusted by doctors through the control units. The supporting units on the device located at the wrist and forearm provide a fulcrum for distal fragment reduction, with no need for a doctor to manually manipulate the fracture fragments. Meanwhile, the device can help doctors maintain the reduction, which is of great importance. The plaster cast was prepared while the device maintained the reduction with absolutely as little movement as possible. Then, the well-molded plaster cast can dorsally buttress the distal fragment in Colles’ fracture and ventrally support the distal fragment in Smith's fracture. After the cast hardens, the device components are removed sequentially. Early redisplacement occurred in 2 of 20 patients (10%) in the device group and 7 of 20 (35%) in the conventional group. Redisplacement remains common after closed reduction and cast immobilization; in the recent multicenter CAST trial, Barvelink et al. reported redisplacement within five weeks in 47% and 49% of patients treated with plaster splints and circumferential casts, respectively (26). Direct comparison with our rates should be made cautiously because the timing of redisplacement assessment differed between studies. The observed absolute difference may be clinically relevant, particularly for older patients who decline surgery or are poor surgical candidates, because preserving an acceptable closed reduction may avoid repeat manipulation or escalation to surgery. Nevertheless, the event counts are small and the study was not powered for this outcome, so the finding should be confirmed in a larger trial.

Several features of the device could contribute to the lower early redisplacement rate observed in the device group. First, the device permits sustained, controlled longitudinal traction during the entire casting process, removing the need for prolonged manual traction which is susceptible to operator fatigue and variability. Second, independent control of ulnar/radial deviation and flexion/extension via the double-plane sliders allows more precise restoration of palmar tilt and ulnar variance—parameters known to influence stability. Third, by maintaining a stable limb position during cast molding, the device improves the fit of the plaster splint and helps create an effective three-point fixation that resists displacement. Finally, the finger-trap style traction distributes force across multiple digits rather than a single point, which may reduce focal pressure and soft-tissue injury risk. Together, these factors plausibly explain the lower early redisplacement observed in our cohort (10% vs. 35%).

Cost is also relevant. At our hospital, surgical treatment cost approximately 30,000–40,000 yuan, whereas device-assisted reduction cost approximately 300 yuan per procedure. These figures suggest potential affordability, particularly in resource-limited settings, but they are local estimates rather than a formal economic analysis. Future studies should compare device acquisition, setup time, staffing, repeat procedures, complications, and downstream treatment costs.

This retrospective study has several limitations. It was conducted at a single center and included only 40 patients identified from an available medical-record cohort, with no *a priori* sample-size calculation; estimates are therefore imprecise and exploratory. Treatment was not randomly assigned, creating a risk of selection bias, and treatment-selection factors may have differed between groups. We did not perform propensity-score adjustment or multivariable control for unmeasured confounding. The retrospective design also depends on the completeness and accuracy of archived records, and blinded outcome assessment could not be confirmed. Pain during the reduction procedure was not systematically assessed using a standardized pain scale and therefore could not be compared between groups. Because patient comfort is an important consideration during closed reduction, prospective studies should incorporate standardized pain assessments during and immediately after the procedure. Preparation time was longer with the device, and structured usability, clinician-satisfaction, and cost-effectiveness assessments were not performed. The study was restricted to adults aged 65 years or older, so findings may not generalize to younger patients with higher-energy injuries. The current device also requires manual adjustment and remains relatively bulky. AI and advanced devices can improve detection and possibly reduction precision, while clinical prediction of redisplacement and robust AI-guided treatment decisions remain an active area needing validation (26, 27). We propose to improve the device to incorporate control by artificial intelligence and to reduce the size and weight to more accurately and intelligently control the device and make it easier to carry and operate. Prospective, registered, multicenter randomized trials with concealed allocation, blinded outcome assessment where feasible, predefined primary outcomes, formal sample-size calculations, and implementation analyses are needed to determine whether the observed associations represent a true treatment effect.

## 5 Conclusion

In this retrospective comparative cohort of adults aged 65 years or older, device-assisted closed reduction was associated with more favorable early radiographic alignment, a lower observed early redisplacement rate, and better 6-month function than conventional manual reduction. Functional differences were attenuated by 1 year. Because treatment was not randomly assigned and residual confounding cannot be excluded, these findings should be interpreted as hypothesis-generating associations rather than definitive evidence of efficacy. The device may be a practical adjunct for maintaining reduction during casting, but its effectiveness requires confirmation in a rigorously designed prospective trial.

## Data Availability

The original contributions presented in the study are included in the article/Supplementary Material, further inquiries can be directed to the corresponding authors.
